# *COL4A6* is dispensable for autosomal recessive Alport syndrome

**DOI:** 10.1038/srep29450

**Published:** 2016-07-05

**Authors:** Tomohiro Murata, Kan Katayama, Toshitaka Oohashi, Timo Jahnukainen, Tomoko Yonezawa, Yoshikazu Sado, Eiji Ishikawa, Shinsuke Nomura, Karl Tryggvason, Masaaki Ito

**Affiliations:** 1 Department of Cardiology and Nephrology, Mie University Graduate School of Medicine, Tsu, Japan; 2 Division of Matrix Biology, Department of Medical Biochemistry and Biophysics, Karolinska Institute, Stockholm, Sweden; 3 Department of Molecular Biology and Biochemistry, Okayama University Graduate School of Medicine, Dentistry and Pharmaceutical Sciences, Okayama, Japan; 4 Department of Pediatric Nephrology and Transplantation, Children’s Hospital, Helsinki University Hospital, Helsinki, Finland; 5 Division of Immunology, Shigei Medical Research Institute, Okayama, Japan

## Abstract

Alport syndrome is caused by mutations in the genes encoding α3, α4, or α5 (IV) chains. Unlike X-linked Alport mice, α5 and α6 (IV) chains are detected in the glomerular basement membrane of autosomal recessive Alport mice, however, the significance of this finding remains to be investigated. We therefore generated mice lacking both α3 and α6 (IV) chains and compared their renal function and survival with *Col4a3* knockout mice of 129 × 1/Sv background. No significant difference was observed in the renal function or survival of the two groups, or when the mice were backcrossed once to C57BL/6 background. However, the survival of backcrossed double knockout mice was significantly longer than that of the mice of 129 × 1/Sv background, which suggests that other modifier genes were involved in this phenomenon. In further studies we identified two Alport patients who had a homozygous mutation in intron 46 of *COL4A4*. The α5 and α6 (IV) chains were focally detected in the glomerular basement membrane of these patients. These findings indicate that although α5 and α6 (IV) chains are induced in the glomerular basement membrane in autosomal recessive Alport syndrome, their induction does not seem to play a major compensatory role.

Type IV collagen, which is a major constituent of basement membranes, consists of six chains from α1 to α6 that are encoded by the *COL4A1* to *COL4A6* genes, respectively. These chains assemble into three types of triple-helical molecules which have chain compositions of α1.α1.α2 (IV), α3.α4.α5 (IV), and α5.α5.α6 (IV)^1^. These protomers dimerize through the noncollagenous 1 (NC1) domains at the carboxyl terminus and form tetramers through their amino terminal domains^2^. In healthy humans, the glomerular basement membrane (GBM) is composed of only the α1.α1.α2 (IV) isoform during fetal development, however, the majority of this isoform is subsequently replaced by the α3.α4.α5 (IV) isoform at the capillary loop stage^3^,^4^.

Alport syndrome (AS), which is caused by mutations in either the *COL4A3*, *COL4A4*, or *COL4A5* gene, is a progressive hereditary nephritic disease that leads to irreversible renal failure^5^. A more effective treatment is currently being sought worldwide because the only established treatment for AS is renal transplantation^6^. X-linked AS (XLAS), which is caused by abnormalities in *COL4A5*, accounts for approximately 80% of AS cases^7^. Autosomal recessive AS (ARAS), which is caused by a defective *COL4A3* or *COL4A4*, accounts for approximately 15%^8^.

*Col4a3* −/− mice are widely used to study AS treatments^9^,^10^ because the degree of renal impairment is highly uniform in comparison to *Col4a5* −/− male mice, which have lifespans that range from 6 to 34 weeks of age^11^. Thus far, numerous drugs have been reported to prolong the lifespan of *Col4a3* −/− mice^12^,^13^,^14^,^15^,^16^.

The pathophysiologies of XLAS and ARAS are considered to be similar because the lack of one subunit of the α3.α4.α5 (IV) isoform results in the degradation of the other two due to a failure to assemble a triple-helical protomer^17^. However, both α1.α1.α2 (IV) and α5.α5.α6 (IV) protomers can theoretically be formed in ARAS, but not in XLAS (in which only α1.α1.α2 (IV) protomer can be formed). In fact, the α5 (IV) and α6 (IV) chains have been identified in Bowman’s capsule (BC) of ARAS patients^18^,^19^. Interestingly, both of these chains were expressed in the GBMs of a canine model of ARAS^20^, and in *Col4a3* −/− mice. In this latter study, affinity fractionation determined that the α5.α5.α6 (IV) isoform formed a network with the α1.α1.α2 isoform^21^. It remains unknown whether the regulation of type IV collagen isoforms in humans differs from that in dogs or mice, since the presence of the α5 and α6 (IV) chains has not been clearly demonstrated in the GBM of ARAS patients.

In XLAS, the GBM, which is composed of only the α1.α1.α2 (IV) protomers, is considered to be more susceptible to proteolytic attack because the α1.α1.α2 (IV) isoform contains fewer disulfide cross-links than the α3.α4.α5 (IV) isoform^3^,^22^. On the other hand, the GBM of *Col4a3* −/− mice is composed of both the α1.α1.α2 (IV) and α5.α5.α6 (IV) protomers, while that of *Col4a5* −/− mice is composed of only the α1.α1.α2 (IV) protomers^21^. Curiously, there is a significant difference in the lifespans of *Col4a3* −/− mice between the 129 × 1/Sv and C57BL/6 strains^23^,^24^. This may be associated with a higher expression of the α5 and α6 (IV) chains in the GBM in the C57BL/6 strain in comparison to that in the 129 × 1/Sv strain^21^.

*COL4A6* is located on the X chromosome in a head-to-head manner with *COL4A5* closely^25^. Although the deletion of both *COL4A5* and *COL4A6* is known to cause AS with diffuse leiomyomatosis^26^,^27^, no study has so far found that a *COL4A6* mutation alone causes hereditary nephropathy.

In the present study, we investigated the role of the α5.α5.α6 (IV) isoform in the GBM on kidney function through the use of mice that lacked both the α3 and α6 (IV) chains.

## Results

### The survival time of Col4a3 −/− mice (3KO) did not become shorter after ablating Col4a6

**Figure 1:**
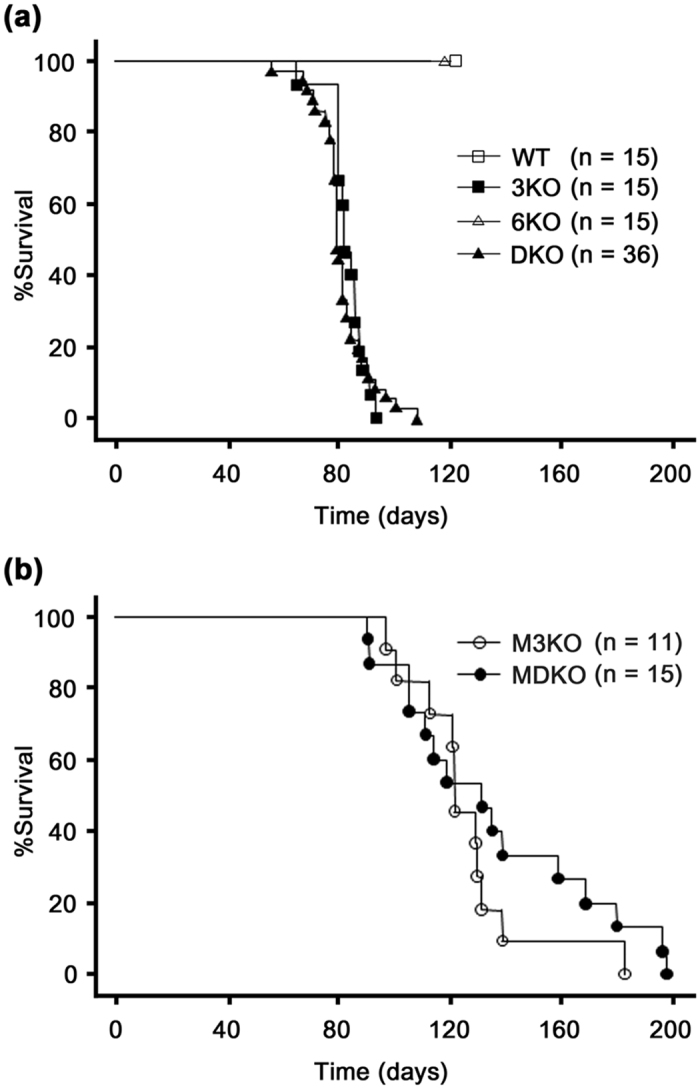
The survival time of *Col4a3* −/− (3KO) mice did not become shorter after ablating *Col4a6*. (**a**) There was no significant difference in the mean survival times of 3KO and *Col4a3 & 6* −/− (DKO) mice (83 ± 6 and 81 ± 9 days, respectively). (**b**) After backcrossing with the C57BL/6 strain once, the survival times of mixed 3KO (M3KO) and mixed DKO (MDKO) mice were 126 ± 23 and 136 ± 36 days, respectively. Although the survival times of M3KO and MDKO mice were significantly improved in comparison to 3KO and DKO mice (log-rank test for trend, *P* < 0.05 for each), there was no significant difference in the mean survival times of M3KO and MDKO mice. WT, wild mice, 6KO, *Col4a6* −/− mice.

The survival times of 3KO and *Col4a3 & 6* −/− mice (DKO) on the 129 × 1/Sv background were 83 ± 6 and 81 ± 9 days, respectively. There was no significant difference in the mean survival times between 3KO and DKO mice (Fig. 1a). Additionally, none of wild mice (WT) and *Col4a6* −/− mice (6KO) in this background died within 120 days. After backcrossing once with the C57BL/6 strain, the survival times of mixed 3KO (M3KO) and mixed DKO (MDKO) mice were 126 ± 23 and 136 ± 36 days, respectively. Although the survival times of M3KO and MDKO mice significantly improved in comparison to those of 3KO and DKO mice (*P* < 0.05 for each), there was no significant difference in the mean survival times between M3KO and MDKO mice (Fig. 1b).

### The blood test results were comparable between 3KO and DKO mice

**Figure 2:**
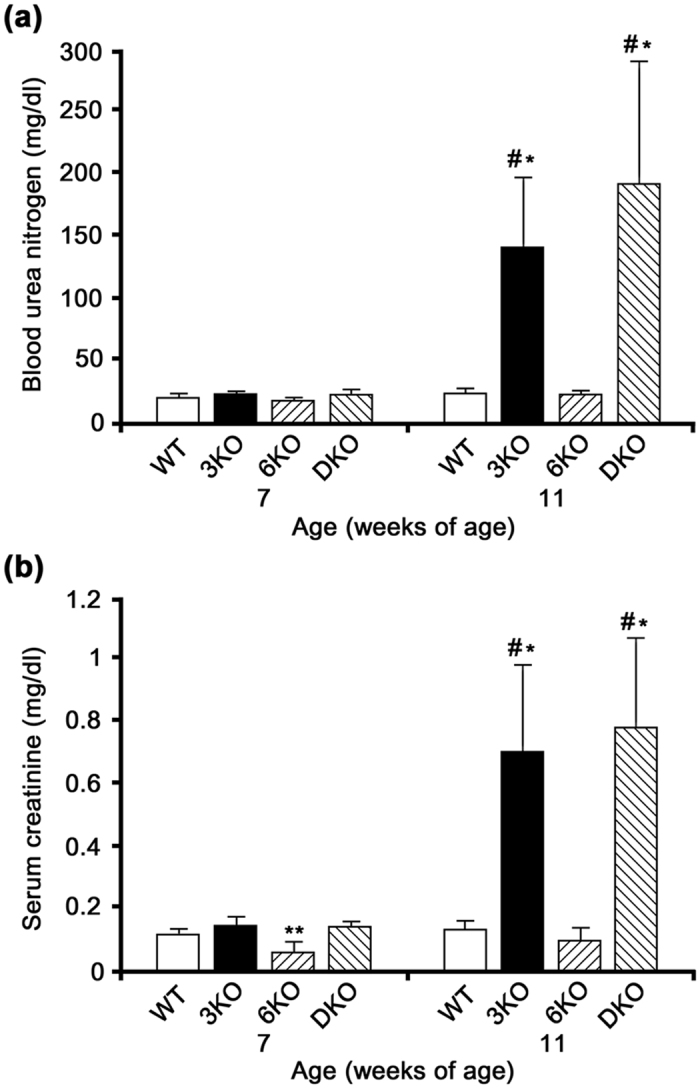
The blood test results were comparable between *Col4a3* −/− (3KO) and *Col4a3 & 6* −/− (DKO) mice. (**a**) No significant differences were noted in the blood urea nitrogen (BUN) levels of the 3KO, 6KO, and DKO mice from those of the WT mice at 7 weeks of age. The BUN levels of 3KO and DKO mice were significantly elevated in comparison to those of wild mice (WT) and *Col4a6* −/− mice (6KO) at 11 weeks of age (^#^*P* < 0.05 versus WT, *P < 0.05 versus 6KO). (**b**) Although no significant differences were noted in the serum creatinine (Cr) levels of the 3KO and DKO mice from that of the WT mice, the Cr level of the 6KO mice was significantly lower than that of the WT mice at 7 weeks of age (**P < 0.05). At 11 weeks of age, the Cr levels of 3KO and DKO mice were significantly elevated in comparison to those of WT and 6KO mice (^#^*P* < 0.05 versus WT, *P < 0.05 versus 6KO). There was no significant difference in the BUN and Cr levels of 3KO and DKO mice at 7 and 11 weeks of age.

The blood urea nitrogen (BUN) and serum creatinine (Cr) levels are summarized in Supplementary Table S1. At 7 weeks of age, there were no significant differences in the BUN levels of 3KO, 6KO, and DKO mice from those of WT mice (Fig. 2a). While we noted no significant differences in the Cr levels of 3KO and DKO from those of WT mice, the Cr level of 6KO mice was significantly lower than that of WT mice (*P* < 0.05, Fig. 2b). At 11 weeks of age, the BUN and Cr levels of 3KO and DKO mice were significantly elevated in comparison to those of WT and 6KO mice (*P* < 0.05, Fig. 2a,b). There was no significant difference in the BUN and Cr levels between 3KO and DKO mice at 7 and 11 weeks of age. At 7 weeks of age, there were no significant differences in the BUN or Cr levels between M3KO and MDKO mice (Supplementary Fig. S1).

### The renal histology was similar between 3KO and DKO mice

**Figure 3:**
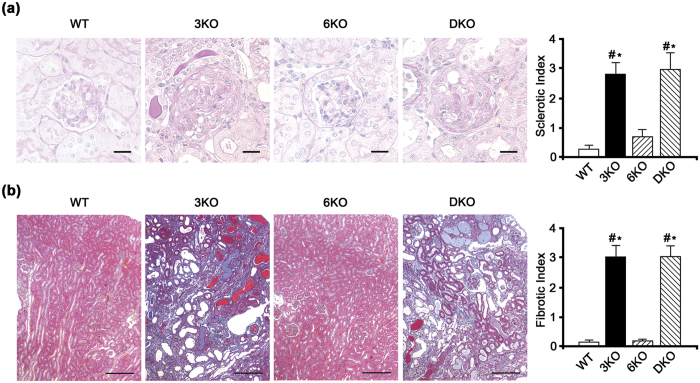
The renal histology was similar between *Col4a3* −/− (3KO) and *Col4a3 & 6* −/− (DKO) mice. (**a**) Although the sclerotic indices of 3KO and DKO mice were significantly increased in comparison to wild mice (WT) and *Col4a6* −/− mice (6KO) (^#^*P* < 0.05 versus WT, *P < 0.05 versus 6KO), there was no significant difference between 3KO and DKO mice at 11 weeks of age. Scale bars, 20 μm. (**b**) Although the fibrotic indices of 3KO and DKO mice were significantly increased in comparison to WT and 6KO mice (^#^*P* < 0.05 versus WT, *P < 0.05 versus 6KO), there was no significant difference between 3KO and DKO mice at 11 weeks of age. Scale bars, 100 μm.

The sclerotic and fibrotic indices of WT, 3KO, 6KO, and DKO mice are summarized in Supplementary Table S2. At 7 weeks of age, the sclerotic indices of 3KO and DKO mice were significantly higher than that of 6KO mice (*P* < 0.05, Supplementary Fig. S2). Although the sclerotic indices of 3KO and DKO mice significantly increased in comparison to WT and 6KO mice at 11 weeks of age (*P* < 0.05), there was no significant difference between 3KO and DKO mice (Fig. 3a). Additionally, although the fibrotic indices of 3KO and DKO mice significantly increased in comparison to WT and 6KO mice (*P* < 0.05), there was no significant difference between 3KO and DKO mice (Fig. 3b). An electron microscopic examination at 11 weeks of age showed that the GBM of 6KO mice was unaffected while that of DKO mice exhibited typical changes of AS (Supplementary Fig. S3).

### The expression α5 (IV) and α6 (IV) chains in the GBM of 3KO mice was not observed after ablating Col4a6

**Figure 4:**
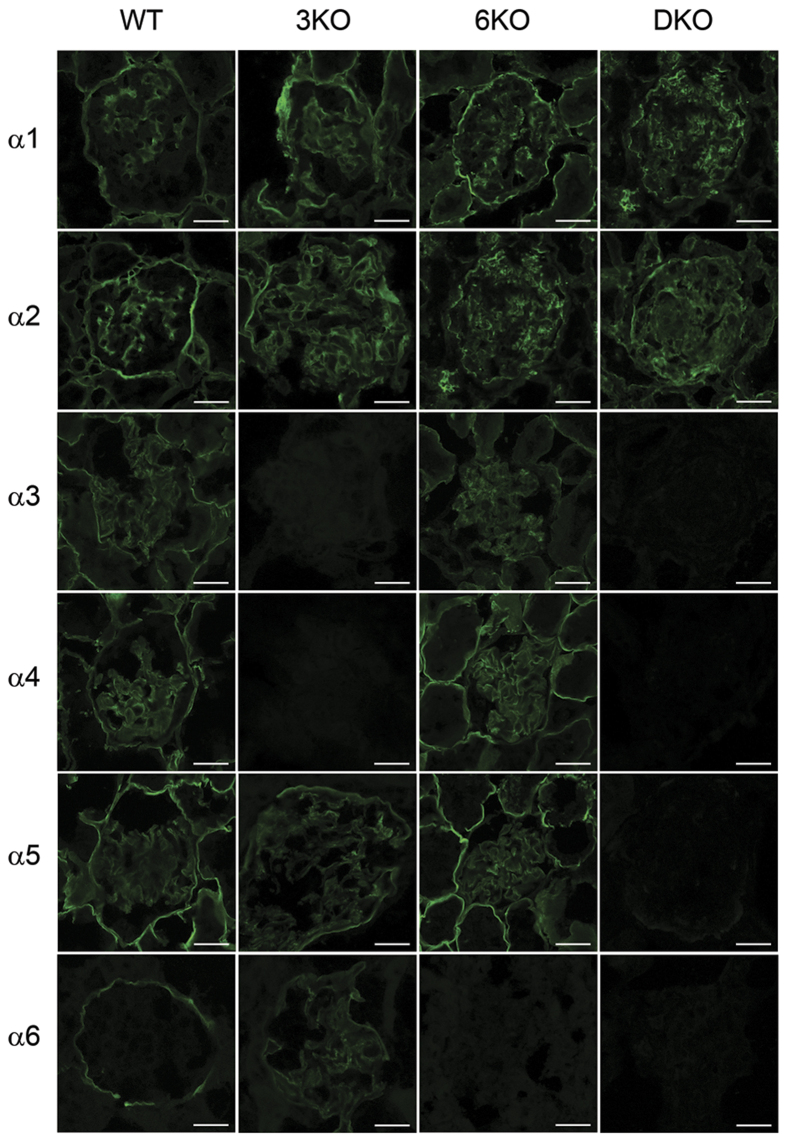
The expression of α5 (IV) and α6 (IV) chains in the glomerular basement membrane (GBM) of *Col4a3* −/− (3KO) mice was not observed after ablating *Col4a6*. α1 and α2 staining of the GBM was positive in the 4 groups at 11 weeks of age. In contrast, α3 and α4 staining of the GBM was positive in wild mice (WT) and *Col4a6* −/− mice (6KO) and negative in 3KO and *Col4a3 & 6* −/− mice (DKO). α5 staining of the GBM was positive in WT and 6KO mice, weakly positive in 3KO mice, and negative in DKO mice. α6 staining of Bowman’s capsule (BC) and the GBM were positive and negative, respectively, in WT mice, while in 3KO mice, both the GBM and BC were positively stained. In 6KO and DKO mice, both the GBM and BC were negatively stained. Scale bars, 20 μm.

At 11 weeks of age, α1 and α2 staining of the GBM was positive in all four groups (Fig. 4). In contrast, α3 and α4 staining of the GBM was positive in WT and 6KO mice and negative in 3KO and DKO mice. α5 staining of the GBM was positive in WT and 6KO mice, weakly positive in 3KO mice, and negative in DKO mice. In WT mice, BC exhibited positivity for α6, while both BC and the GBM of 3KO mice were positively stained. In contrast, α6 staining of BC and the GBM was completely absent in 6KO and DKO mice. These results were compatible with the fact that both the α3.α4.α5 (IV) and α5.α5.α6 (IV) protomers are absent from the GBM of DKO mice.

### α3 (IV) to α6 (IV) chains were negative in the total kidney lysate of DKO mice

**Figure 5:**
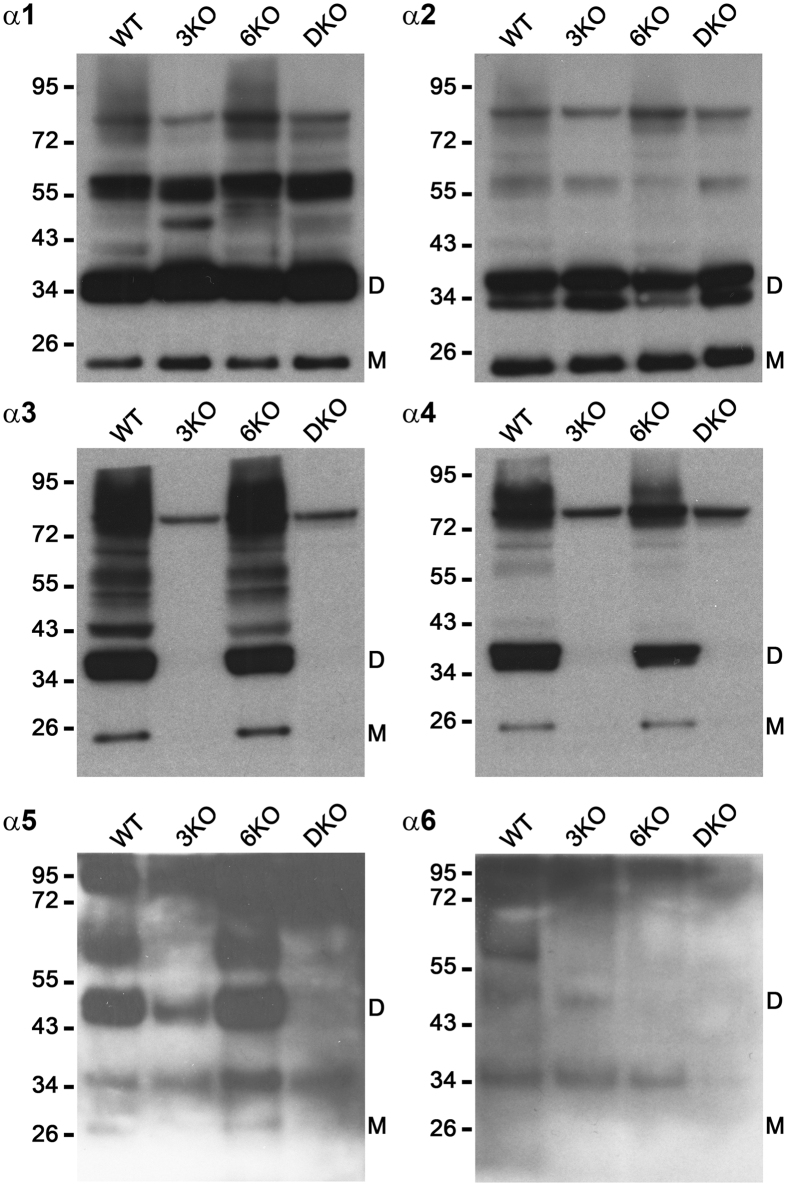
α3 (IV) to α6 (IV) chains were negative in the total kidney lysate of DKO mice. Type IV collagen α1 and α2 chains were detected as a monomer and a dimer, respectively, in all 4 groups, although type IV collagen α3 and α4 chains were only detected in wild mice (WT) and *Col4a6* −/− mice (6KO) at 11 weeks of age. The dimer of the α5 (IV) chains was detected in the WT and 6KO mice and weakly detected in the *Col4a3* −/− mice (3KO) but undetectable in the *Col4a3 & 6* −/− mice (DKO). The monomer of the α5 (IV) chains was detected in the WT and 6KO mice but undetectable in the 3KO and DKO mice. The dimer of the α6 (IV) chains was weakly detected in the WT and 3KO mice but undetectable in the 6KO and DKO mice. D, noncollagenous domain 1 (NC1) dimer; M, NC1 monomer.

A Western blot analysis detected the α1 (IV) and α2 (IV) chains as a monomer and a dimer, respectively, in all four groups, whereas the α3 (IV) and α4 (IV) chains were only detected in WT and 6KO mice at 11 weeks of age (Fig. 5). The dimer of the α5 (IV) chains was detected in the WT and 6KO mice and weakly detected in the 3KO mice but undetectable in DKO mice. The monomer of the α5 (IV) chains was detected in the WT and 6KO mice but undetectable in the 3KO and DKO mice. The dimer of the α6 (IV) chains was weakly detected in the WT and 3KO mice but undetectable in the 6KO and DKO mice. Similar results of the α5 (IV) and α6 (IV) chains were obtained from the RIPA buffer-treated samples (Supplementary Fig. S4).

### The Col4a1 and Col4a2 expressions were upregulated in 3KO and DKO mice compared to WT and 6KO mice

**Figure 6:**
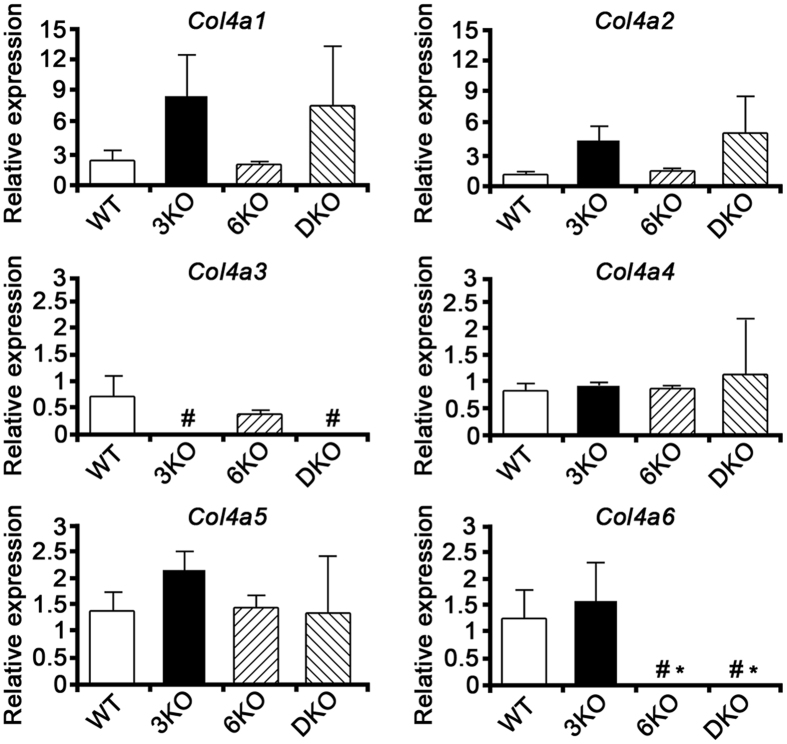
The *Col4a1* and *Col4a2* expressions were upregulated in *Col4a3* −/− mice (3KO) and *Col4a3 & 6* −/− mice (DKO) compared to wild mice (WT) and *Col4a6* −/− mice (6KO). The *Col4a1* expression levels in 3KO and DKO mice were 3.8 and 3.2 times higher, respectively, than that in WT mice. The expression levels of *Col4a2* in 3KO and DKO mice were 4.1 and 4.6 times higher, respectively, than that in WT mice. The expression levels of *Col4a3* in the 3KO and DKO mice were significantly lower than in the WT mice (^#^*P* < 0.05). The expression levels of *Col4a5* in 3KO and DKO mice were 1.6 and 1 times higher, respectively, than that in WT mice. Finally, the expression level of *Col4a6* in 3KO mice was 1.3 times higher than that in WT mice, while the expression levels of *Col4a6* in the 6KO and DKO mice were significantly lower than those of the WT and 3KO mice (^#^*P* < 0.05 versus WT, *P < 0.05 versus 3KO).

The relative expression levels of *Col4a1* to *Col4a6* in WT, 3KO, 6KO, and DKO mice are summarized in Supplementary Table S3. A real-time reverse transcription polymerase chain reaction (RT-PCR) revealed that the relative expression levels of *Col4a1* and *Col4a2* in 3KO and DKO mice were 3.8/3.2 and 4.1/4.6 times higher, respectively, than that in WT mice, whereas the expression level of *Col4a5* was only 1.6 and 1 times higher, respectively (Fig. 6). Furthermore, in 3KO mice, the *Col4a6* expression was only 1.3 times higher than that in WT mice.

### α5 (IV) and α6 (IV) chains were focally detected in the GBM of ARAS patients

**Figure 7:**
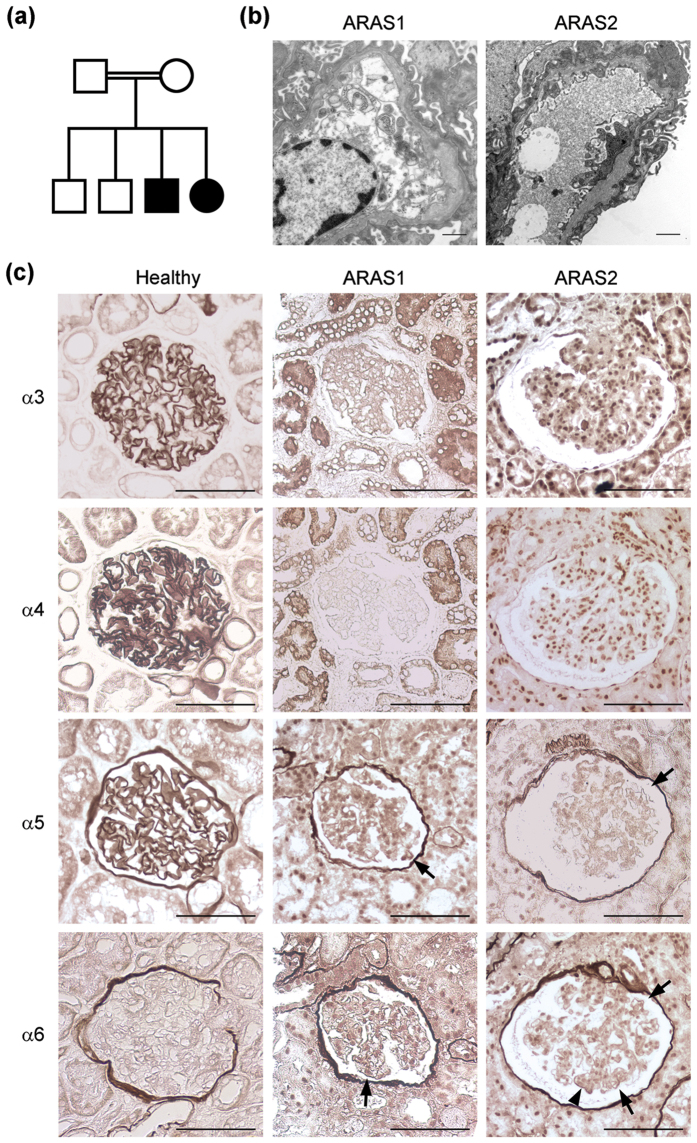
α5 (IV) and α6 (IV) chains were focally detected in the glomerular basement membrane (GBM) of autosomal recessive Alport syndrome (ARAS) patients. (**a**) The pedigree of a Turkish family with consanguineous parents. One son and one daughter of consanguineous Turkish parents had a homozygous mutation (4523-1G > A in intron 46 of *COL4A4*). The parents and two brothers did not present renal impairment. Open square, unaffected male; open circle, unaffected female; close square, affected male; close circle, affected female. (**b**) An electron microscopic analysis showed typical thickening, thinning, and splitting of the GBM in the affected son (ARAS1) and the affected daughter (ARAS2). Scale bars, 1 μm. (**c**) In the healthy subjects, the GBM was positively stained with α3, α4, and α5, while Bowman´s capsule (BC) was positively stained with α5 and α6. In both of the ARAS patients, the GBM was negatively stained with α3 and α4, while BC was positively stained with α5 and α6. Some areas of the GBMs of these patients were positively stained with α5 and α6 (black arrows). In the ARAS2 patient, α6 staining was strong and linear in one area of the GBM (black arrowhead). Scale bars, 100 μm.

Two siblings, a male (ARAS1) and a female (ARAS2), of consanguineous Turkish parents presented with hematuria, proteinuria, and progressive renal insufficiency (Fig. 7a). There were no known kidney diseases in the family. A genomic sequence analysis of the *COL4A3* and *COL4A4* genes in both patients revealed a homozygous mutation (c.4523-1G > A) in intron 46 of the *COL4A4* gene, which resided in the AG consensus splice acceptor. The case histories of these two patients are described in the Supplementary Methods section. An electron microscopic examination showed typical thickening, thinning, and splitting in the GBMs of both patients (Fig. 7b). The GBM of the control samples was positively stained by α3, α4, and α5, while BC was positively stained by α5 and α6 (Fig. 7c). α3 and α4 staining of the GBM was negative in both patients, while α5 and α6 staining of BC was positive. Moreover, α5 and α6 staining of some areas of the GBM was positive in the two patients (arrows and Supplementary Fig. S5). In the ARAS2 patient, strong and linear α6 staining was observed in one region (arrowhead).

## Discussion

In this study, we demonstrated that there was no significant difference in the renal function or the survival between 3KO and DKO mice on the 129 × 1/Sv genetic background, although a weak expression of the α5 and α6 (IV) chains in the GBM of 3KO mice was detected. Additionally, there was no significant difference in the survival between M3KO and MDKO mice after backcrossing once with the C57BL/6 strain. These results indicated that the deposition of the α5.α5.α6 (IV) isoform in the GBM of 3KO mice does not play a major role in extending the survival period. On the contrary, MDKO mice survived longer than 129 × 1/Sv DKO mice, which was compatible with the previous suggestion that there might be modifier genes involved in the longer survival on the C57BL/6 genetic background, compared with the 129 × 1/Sv background, that are irrelevant to the *Col4a6* expression^23^.

Although the survival of *Col4a5* −/− mice on the C57BL/6 background was reported to be 161 days, there are at least two different reports about the survival of C57BL/6 *Col4a3* −/− mice (194 days and 165 days, respectively)^23^,^24^. This indicates that the survival of C57BL/6 *Col4a3* −/− mice is quite variable in each facility and easily influenced by environmental factors, such as breeding conditions. In fact, the survival of *Col4a3* −/− mice on the 129 × 1/Sv background was 83 days in this study, while it was 66 days and 71 days in previous reports^13^,^23^. Therefore, a comparison of the survival between different facilities should be interpreted carefully, and it is better to make a direct comparison in one facility under the same environmental conditions.

According to our immunofluorescence study, the GBM of DKO mice contained only α1 and α2 (IV) chains, whereas that of 3KO mice contained the α1, α2, α5, and α6 (IV) chains. The GBM of WT and 6KO mice contained the α1 to α5 (IV) chains. These results suggested that, in comparison to the GBM of WT and 6KO mice which is comprised of both the α1.α1.α2 (IV) and α3.α4.α5 (IV) protomers, the GBM of DKO mice is composed only of the α1.α1.α2 (IV) protomers, while that of 3KO mice contains low levels of the α5.α5.α6 (IV) protomers in addition to the α1.α1.α2 (IV) protomers. By using real-time quantitative PCR, we attempted to examine the compensatory mechanism among *Col4a1* to *Co4a6* mRNAs. The results indicated that loss of the *Col4a3* expression is compensated mainly by *Col4a1* and *Col4a2* mRNAs, rather than those encoding *Col4a5* and *Col4a6*, which is consistent with previous results^9^.

Furthermore, all of the 6KO mice with an 129 × 1/Sv background examined in this study survived until 120 days, and we observed no significant differences between the WT and 6KO mice at 11 weeks of age in the BUN and Cr levels, and the sclerotic index and fibrotic index. As a preliminary experiment, we examined the urine albumin-to-creatinine ratio in the WT and 6KO mice with a C57BL/6 background and observed no significant differences between the two groups (Supplementary Fig. S6). These data support the lack of any reports of hereditary nephropathy due to *COL4A6* mutation alone. While the continuous deletion of both *COL4A5* and *COL4A6* is known to cause AS with diffuse leiomyomatosis^26^, the 6KO mice did not show any apparent esophageal leiomyomatosis (data not shown). Unlike XLAS, which is related to the loss of the α5 (IV) chains due to mutations in *COL4A5*, leiomyomatosis is considered to be caused by the upregulation of the *IRS4* gene, which is located next to *COL4A5*^28^.

It was of interest to examine the expression of the α5 and α6 (IV) chains in kidney sections of genetically-diagnosed human ARAS patients because these chains are detected in the GBM of ARAS canine and murine models^20^,^21^. Our two ARAS patients were found to have a homozygous point mutation in intron 46 of *COL4A4*, which very likely affects splicing of an exon encoding part of the NC1 domain of α4 (IV) chains, thereby leading to failure of α3.α4.α5 (IV) protomer assembly. The electron microscopy results were compatible with the diagnosis of ARAS. Immunohistochemical staining for the α5 and α6 (IV) chains showed partially positive signals in the GBM of these two patients, while α3 and α4 (IV) staining was negative. These results might indicate that the appearance of the α5 and α6 (IV) chains in the GBM is a phenomenon in ARAS that is common among different species; at the very least, the α5.α5.α6 (IV) protomer did not appear to be evenly distributed in humans.

In conclusion, our study demonstrated that although the α5/α6 (IV) collagen chains are observed in the GBM of 3KO mice, the α5.α5.α6 (IV) isoform does not appear to have a compensatory role. The extension of the survival of backcrossed DKO mice in comparison to 129 × 1/Sv DKO mice strongly suggested the existence of modifier genes other than *Col4a6*. Further investigations are warranted due to the diverse pathophysiology of this hereditary disease.

## Methods

### Animal experimental design

*Col4a3* +/− mice (129-*Col4a3*^*tm1Dec*^/J, stock number 002908) were purchased from the Jackson Laboratory (Bar Harbor, MN, USA)^9^. *Col4a6* +/− mice were provided by Okayama University. Briefly, the *Col4a6* gene was inactivated by replacing parts of exon 2 and intron 2 with a Neomycin cassette^29^. Genotyping primers for 6KO were as follows: A6PA, 5′-ATTGTGGCTGTTCCTGGTTT-3′); A6PB, 5′-CGGAGAGCATTAGGCTAATG-3′; NeoPA, 5′-CTGCTCTTTACTGAAGGCTCTTT-3′. 3KO and 6KO mice were then backcrossed once with C57BL/6 mice and F1 offspring were crossed for the assessment of the survival time. Littermates were used as controls. All experimental protocols were approved by the Animal Care and Use Committee of Mie University (No. 18-8) and all experiments were performed in accordance with the approved guidelines.

### Lifespan

The survival times of WT (*n* = 15), 3KO (*n* = 15), 6KO (*n* = 15), and DKO (*n* = 36) mice were measured until 120 days and evaluated using the Kaplan-Meier method. The survival times of M3KO (*n* = 11) and MDKO (*n* = 15) were also evaluated using the Kaplan-Meier method.

### Blood chemistry analysis

The BUN and Cr levels in the WT, 3KO, 6KO, and DKO mice were examined at 7 and 11 weeks (*n* = 10 each) while those of the M3KO and MDKO mice were examined at 7 weeks (*n* = 5 each). The BUN and Cr levels were measured using a urease and glutamate dehydrogenase assay and an enzymatic assay, respectively, as described previously^30^.

### Morphological evaluation

Periodic acid-Schiff staining was performed using formalin-fixed, paraffin-embedded kidney sections (3 μm) that were harvested at 7 and 11 weeks of age in order to analyze sclerotic changes in the WT, 3KO, 6KO, and DKO mice, except for the 7-week-old WT mice (*n* = 5 each). Masson’s trichrome staining was performed to analyze tubulointerstitial fibrotic changes (*n* = 5 each). Under blinded condition, the sclerotic index was examined in 20 randomly selected glomeruli from each mouse and the fibrotic index, which was used to assess tubulointerstitial damage, was examined in 20 randomly selected areas from each mouse. The sclerotic and fibrotic indices were divided into five categories: 0 (no apparent damaged area), +1 (1–25% damaged area), +2 (26–50% damaged area), +3 (51–75% damaged area), and +4 (76–100% damaged area) as described previously^31^.

### The immunofluorescence study of the α1 – α6 (IV) chains

Cryosections (4 μm) were collected from 11-week-old mice (n = 3 per group) and silanized slides were prepared. The slides were incubated in acetone for 10 minutes at −20 °C, followed by incubation with primary antibodies for 60 minutes at 4 °C overnight after blocking with 10% normal goat serum for 30 minutes at room temperature. With the exception of H22, monoclonal primary antibodies specific to the NC1 domains of type IV collagen (α1 [H11], α2 [H22], α3 [H31], α4 [RH42], α5 [b14], and α6 [B66]) were diluted to 1:100; H22 was diluted to 1:50^32^. H11-, H22-, H31-, and b14-stained sections were treated with 6 M urea in 0.05 M glycine/HCl (pH 3.5) for 10 minutes and B66 for 1 min before blocking. The specimens were incubated with Alexa Fluor 488 goat anti-rat IgG (H + L) (Thermo Fisher Scientific) at 1:1000 dilution for one hour at room temperature. The primary antibodies were omitted in the negative controls. All images were taken by confocal microscopy using a Zeiss LSM 700 microscope (Zeiss, Oberkochen, Germany).

### Western blotting

The minced mouse kidneys of the four groups (*n* = 3 each) were incubated at 37 °C for 24 hours with 0.5 mg of collagenase I (Worthington Biochemical Corporation, Lakewood, NJ, USA) and 2 volumes of digestion buffer (0.05M HEPES, pH 7.5, 0.01 M CaCl_2_, 4 mM N-ethylmaleimide, 1 mM phenylmethanesulfonyl fluoride, 5 mM benzamidine HCl, and 25 mM 6-aminohexanoic acid) to solubilize the NC1 hexamers of type IV collagen. To detect the α5 (IV) and α6 (IV) chains, the minced kidneys were also homogenized in RIPA buffer (50 mM Tris-HCl, pH 7.5, 0.15 M NaCl, 1 mM ethylenediaminetetraacetic acid, 1% NP-40, 0.1% sodium deoxycholate, 0.1% sodium dodecyl sulfate) with cOmplete protease inhibitor (Roche Life Science) and then were incubated on ice for 10 minutes. After centrifugation, the pellets were incubated in 2 volumes of digestion buffer with 0.5 mg of collagenase I at 37 °C for 24 hours. Polyacrylamide gel electrophoresis was performed on 4–12% gels under non-reducing conditions by applying 5 μg of kidney lysates from 11-week-old mice. The gels were transferred to polyvinylidene difluoride membranes and the membranes were incubated with primary antibodies at room temperature for 1 hour after blocking. H11, H22, H31, and RH42 were diluted to 1:100 and M54 for mouse α5 (IV) and M69 for mouse α6 (IV) were diluted to 1:50 as the primary antibodies. Horseradish peroxidase-linked secondary anti-rat antibodies were diluted to 1:3000. Immunodetection was performed using an enhanced chemiluminescence kit (Amersham Biosciences). The blots were then exposed to film for various lengths of time.

### Real-time RT-PCR

Total RNA was isolated from the frozen kidneys of 11-week-old mice (*n* = 3 each) using TRIzol reagent (Thermo Fisher Scientific) and RT was carried out at 50 °C for 60 minutes in 20 μl of RT mixture containing 5 μg of total RNA, SuperScript III (Thermo Fisher Scientific), and oligo dT primers. The primer sequences were as follows: *Col4a1*, 5′-ATGCCCTTTCTCTTCTGCAA-3′ (forward) and 5′-ACTGCGGAATCTGAATGGTC-3′ (reverse); *Col4a2*, 5′-GTGCACAGCCAGGATACCTC-3′ (forward) and 5′-CCCCCGTTACACTCGATAAA-3′ (reverse); *Col4a3*, 5′-AACAAGGGCTTGAAAGGACA-3′ (forward) and 5′-TCTTGTCCATGTGCACGTTT-3′ (reverse); *Col4a4*, 5′-TTCCTCCTGGTTCTCCACAG-3′ (forward) and 5′-TGGATGTTGCAGTAGGCAAA-3′ (reverse); *Col4a5*, 5′-TGCCTTTCATGTTCTGCAAC-3′ (forward) and 5′-CCCTGAGGACAGTGTGGAAT-3′ (reverse); *Col4a6*, 5′-GAACAAACTGCCAAGCATCA-3′ (forward) and 5′-CTCCCACATGGCTTTCCATA-3′ (reverse); *Gapdh*, 5′-CGTCCCGTAGACAAAATGGT-3′ (forward) and 5′-GAATTTGCCGTGAGTGGAGT-3′ (reverse). For each sample, quantitative PCR was performed in triplicate using Power SYBR Green Master Mix (Thermo Fisher Scientific) with an ABI Prism 7300 Real-time PCR system (Applied Biosystems). The relative expression levels were calculated with the δ-δ cycle threshold (Ct) relative quantification (RQ) method (*ddCt*, *RQ* = 2^*−ddCt*^).

### Case reports of the ARAS patients

Written informed consent was obtained from both of the patients and all clinical experiments were performed in accordance with the Declaration of Helsinki. The case histories of these two patients are described in the Supplementary Methods section.

### Immunohistochemistry of formalin-fixed paraffin-embedded human kidney sections

Normal human kidney paraffin sections were purchased from Zyagen Laboratories. Antigen retrieval was performed according to the methods of previous reports with minor modifications^33^,^34^. Briefly, after deparaffinization, the specimens were incubated in 0.2 M HCl and autoclaved at 121 °C for 6 minutes. The primary antibodies against the NC1 domains of α3, α4, α5, and α6 (IV) chains were H31, H43, H52, and H63, respectively. All of the antibodies were diluted to 1:5 and the sections were incubated at 4 °C overnight. A VECTASTAIN Elite ABC kit (Vector Laboratories) was used according to the manufacturer’s instructions. 3, 3′-Diaminobenzidine staining was performed at room temperature for 5 minutes. Sections from the same glomerulus of the ARAS patients were assessed. The images were captured using the Spot software program (version 5.0) through a Nikon Eclipse E800 microscope.

### Statistical analysis

The data are expressed as the mean ± SD. The Kaplan-Meier method was used to compare the survival time and a log-rank test for trend was used to compare the survival curves among more than two groups. Two-way analysis of variance (ANOVA) was performed to analyze the BUN and Cr data. One-way ANOVA was performed, followed by the Tukey-Kramer post-hoc test, to analyze the other data. *P* values of <0.05 were considered to indicate statistical significance. The StatView software package (version 5.0, SAS statistical software) was used to perform all of the statistical analyses.

## Additional Information

**How to cite this article**: Murata, T. *et al*. *COL4A6* is dispensable for autosomal recessive Alport syndrome. *Sci. Rep.* **6**, 29450; doi: 10.1038/srep29450 (2016).

## Supplementary Material

Supplementary Information

## References

[b1] Borza D. B. . The NC1 domain of collagen IV encodes a novel network composed of the alpha 1, alpha 2, alpha 5, and alpha 6 chains in smooth muscle basement membranes. J. Biol. Chem. 276, 28532–28540 (2001). 11375996 10.1074/jbc.M103690200

[b2] Timpl R., Wiedemann H., van Delden V., Furthmayr H. & Kühn K. A network model for the organization of type IV collagen molecules in basement membranes. Eur. J. Biochem. 120, 203–211 (1981). 6274634 10.1111/j.1432-1033.1981.tb05690.x

[b3] Kalluri R., Shield C. F., Todd P., Hudson B. G. & Neilson E. G. Isoform switching of type IV collagen is developmentally arrested in X-linked Alport syndrome leading to increased susceptibility of renal basement membranes to endoproteolysis. J. Clin. Invest. 99, 2470–2478 (1997). 9153291 10.1172/JCI119431 PMC508088

[b4] Miner J. H. & Sanes J. R. Collagen IV alpha 3, alpha 4, and alpha 5 chains in rodent basal laminae: sequence, distribution, association with laminins, and developmental switches. J. Cell Biol. 127, 879–891 (1994). 7962065 10.1083/jcb.127.3.879 PMC2120241

[b5] Gubler M. . Alport’s syndrome. A report of 58 cases and a review of the literature. Am. J. Med. 70, 493–505 (1981). 7211891 10.1016/0002-9343(81)90571-4

[b6] Katayama K., Nomura S., Tryggvason K. & Ito M. Searching for a treatment for Alport syndrome using mouse models. World J. Nephrol. 3, 230–236 (2014). 25374816 10.5527/wjn.v3.i4.230 PMC4220355

[b7] Flinter F. A., Cameron J. S., Chantler C., Houston I. & Bobrow M. Genetics of classic Alport’s syndrome. Lancet. 2, 1005–1007 (1988). 2902439 10.1016/s0140-6736(88)90753-2

[b8] Mochizuki T. . Identification of mutations in the alpha 3(IV) and alpha 4(IV) collagen genes in autosomal recessive Alport syndrome. Nat. Genet. 8, 77–81 (1994). 7987396 10.1038/ng0994-77

[b9] Cosgrove D. . Collagen COL4A3 knockout: a mouse model for autosomal Alport syndrome. Genes Dev. 10, 2981–2992 (1996). 8956999 10.1101/gad.10.23.2981

[b10] Miner J. H. & Sanes J. R. Molecular and functional defects in kidneys of mice lacking collagen alpha 3(IV): implications for Alport syndrome. J. Cell Biol. 135, 1403–1413 (1996). 8947561 10.1083/jcb.135.5.1403 PMC2121079

[b11] Rheault M. N. . Mouse model of X-linked Alport syndrome. J. Am. Soc. Nephrol. 15, 1466–1474 (2004). 15153557 10.1097/01.asn.0000130562.90255.8f

[b12] Gross O. . Nephroprotection by antifibrotic and anti-inflammatory effects of the vasopeptidase inhibitor AVE7688. Kidney Int. 68, 456–463 (2005). 16014022 10.1111/j.1523-1755.2005.00423.x

[b13] Gross O. . Preemptive ramipril therapy delays renal failure and reduces renal fibrosis in COL4A3-knockout mice with Alport syndrome. Kidney Int. 63, 438–446 (2003). 12631109 10.1046/j.1523-1755.2003.00779.x

[b14] Gross O. . Antifibrotic, nephroprotective potential of ACE inhibitor vs AT1 antagonist in a murine model of renal fibrosis. Nephrol. Dial. Transplant. 19, 1716–1723 (2004). 15128880 10.1093/ndt/gfh219

[b15] Koepke M. L. . Nephroprotective effect of the HMG-CoA-reductase inhibitor cerivastatin in a mouse model of progressive renal fibrosis in Alport syndrome. Nephrol. Dial. Transplant. 22, 1062–1069 (2007). 17287218 10.1093/ndt/gfl810

[b16] Ninichuk V. . Delayed chemokine receptor 1 blockade prolongs survival in collagen 4A3-deficient mice with Alport disease. J. Am. Soc. Nephrol. 16, 977–985 (2005). 15716328 10.1681/ASN.2004100871

[b17] Boutaud A. . Type IV collagen of the glomerular basement membrane. Evidence that the chain specificity of network assembly is encoded by the noncollagenous NC1 domains. J. Biol. Chem. 275, 30716–30724 (2000). 10896941 10.1074/jbc.M004569200

[b18] Gubler M. C. . Autosomal recessive Alport syndrome: immunohistochemical study of type IV collagen chain distribution. Kidney Int. 47, 1142–1147 (1995). 7783412 10.1038/ki.1995.163

[b19] Nomura S. . Molecular genetic and immunohistochemical study of autosomal recessive Alport’s syndrome. Am. J. Kidney Dis. 31, E4 (1998). 10074584 10.1053/ajkd.1998.v31.pm10074584

[b20] Lees G. E. . A model of autosomal recessive Alport syndrome in English cocker spaniel dogs. Kidney Int. 54, 706–719 (1998). 9734596 10.1046/j.1523-1755.1998.00062.x

[b21] Kang J. S. . Loss of alpha3/alpha4(IV) collagen from the glomerular basement membrane induces a strain-dependent isoform switch to alpha5alpha6(IV) collagen associated with longer renal survival in Col4a3−/− Alport mice. J. Am. Soc. Nephrol. 17, 1962–1969 (2006). 16769745 10.1681/ASN.2006020165

[b22] Gunwar S. . Glomerular basement membrane. Identification of a novel disulfide-cross-linked network of alpha3, alpha4, and alpha5 chains of type IV collagen and its implications for the pathogenesis of Alport syndrome. J. Biol. Chem. 273, 8767–8775 (1998). 9535854 10.1074/jbc.273.15.8767

[b23] Andrews K. L., Mudd J. L., Li C. & Miner J. H. Quantitative trait loci influence renal disease progression in a mouse model of Alport syndrome. Am. J. Pathol. 160, 721–730 (2002). 11839593 10.1016/S0002-9440(10)64892-4 PMC1850644

[b24] Gross O. . Stem cell therapy for Alport syndrome: the hope beyond the hype. Nephrol. Dial. Transplant. 24, 731–734 (2009). 19110486 10.1093/ndt/gfn722 PMC3888105

[b25] Sugimoto M., Oohashi T. & Ninomiya Y. The genes COL4A5 and COL4A6, coding for basement membrane collagen chains alpha 5(IV) and alpha 6(IV), are located head-to-head in close proximity on human chromosome Xq22 and COL4A6 is transcribed from two alternative promoters. Proc. Natl. Acad. Sci. USA. 91, 11679–11683 (1994). 7972123 10.1073/pnas.91.24.11679 PMC45295

[b26] Zhou J. . Deletion of the paired alpha 5(IV) and alpha 6(IV) collagen genes in inherited smooth muscle tumors. Science. 261, 1167–1169 (1993). 8356449 10.1126/science.8356449

[b27] Oohashi T. . Clonal overgrowth of esophageal smooth muscle cells in diffuse leiomyomatosis-Alport syndrome caused by partial deletion in COL4A5 and COL4A6 genes. Matrix Biol. 30, 3–8 (2011). 20951201 10.1016/j.matbio.2010.09.003

[b28] Mehine M. . Characterization of uterine leiomyomas by whole-genome sequencing. N. Engl. J. Med. 369, 43–53 (2013). 23738515 10.1056/NEJMoa1302736

[b29] Fox M. A. . Distinct target-derived signals organize formation, maturation, and maintenance of motor nerve terminals. Cell. 129, 179–193 (2007). 17418794 10.1016/j.cell.2007.02.035

[b30] Katayama K. . Irradiation prolongs survival of Alport mice. J. Am. Soc. Nephrol. 19, 1692–700 (2008). 18480315 10.1681/ASN.2007070829 PMC2518432

[b31] Chatziantoniou C., Boffa J. J., Ardaillou R. & Dussaule J. C. Nitric oxide inhibition induces early activation of type I collagen gene in renal resistance vessels and glomeruli in transgenic mice. Role of endothelin. J. Clin. Invest. 101, 2780–2789 (1998). 9637712 10.1172/JCI2132 PMC508869

[b32] Sado Y. . Establishment by the rat lymph node method of epitope-defined monoclonal antibodies recognizing the six different alpha chains of human type IV collagen. Histochem. Cell Biol. 104, 267–275 (1995). 8548560 10.1007/BF01464322

[b33] Naito I. . X-linked Alport syndrome with normal distribution of collagen IV alpha chains in epidermal basement membrane. Contrib. Nephrol. 122, 134–139 (1997). 9399056 10.1159/000059891

[b34] Naito I., Ninomiya Y. & Nomura S. Immunohistochemical diagnosis of Alport’s syndrome in paraffin-embedded renal sections: antigen retrieval with autoclave heating. Med. Electron Microsc. 36, 1–7 (2003). 12658346 10.1007/s007950300000

