# Supplementary material for: COL4A6 is dispensable for autosomal recessive Alport syndrome
Source: Sci Rep. 2016 Jul 5;6:29450. doi: 10.1038/srep29450 (PMC4932521; doi:10.1038/srep29450)
Supplement: Supplementary Information [file srep29450-s1.pdf]

Supplementary Information

COL4A6 is dispensable for autosomal recessive Alport syndrome

Tomohiro Murata[1,+], Kan Katayama[1,2,+,*], Toshitaka Oohashi[3], Timo Jahnukainen[4], Tomoko Yonezawa[3], Yoshikazu Sado[5], Eiji Ishikawa[1], Shinsuke Nomura[1], Karl Tryggvason[2], Masaaki Ito[1]

Supplementary Methods

Case 1

The first patient (ARAS1) presented at 7 years of age with microscopic hematuria and proteinuria in January 2001. He was first examined in a routine health checkup. *Streptococcus pyogenes* was detected in a throat culture and post-infectious glomerulonephritis was suspected. His proteinuria gradually increased and a renal biopsy was performed in January 2002. The biopsy showed slight mesangial proliferation and IgM positivity. An electron microscopic study was not performed. He was put on daily enalapril medication. He frequently suffered from streptococcal tonsillitis and tonsillectomy was performed in December 2004. He subsequently developed moderate hearing loss (55 dB at 2,000 Hz). A kidney biopsy was repeated after referral to pediatric unit in 2007 due to the suspicion of AS. His renal function remained normal until 2009. His glomerular filtration rate (mGFR), which was 110 ml/min/1.73 m^2 in October 2007, gradually became impaired. By October 2010, his mGFR was 40 ml/min/1.73 m^2. In February 2012, his plasma creatinine (PCr) suddenly elevated to 11.37 mg/dL and hemodialysis was started. A kidney transplant from his mother was performed in September 2012. One year after transplantation, his PCr was 1.07 mg/dL. He had microscopic hematuria (79 x 10^6/L), but no proteinuria. He has had neither microscopic hematuria nor proteinuria since February 2015.

Case 2

The second patient (ARAS2), who was the younger (by one year) sister of ARAS1, presented with macroscopic hematuria at 6 years of age in January 2001. Similar to her brother, she also had *Streptococcus pyogenes* in her throat culture. Both proteinuria and microscopic hematuria, however, persisted and a renal biopsy was performed 8 months later. The biopsy finding was identical to that of her brother's, and she was treated with enalapril (0.5 mg/kg/d) and followed up by a pediatrician. Her PCr and BUN levels were normal.

After a follow-up in August 2007, she was transferred to Helsinki University Hospital. Her mGFR was 85 ml/min/1.73m^2, and her PCr and BUN levels were 0.38 mg/dL, and 2.29 mg/dL, respectively. Her urinary protein level had increased to 1.6 g/L (protein creatinine ratio 4,300 mg/g) and her urine erythrocyte count was 49 x 10^6/L. She had developed moderate sensorineural hearing impairment (60 dB at 2,000 Hz). Optical coherence tomography revealed foveal atrophy and thinning. Her brother had presented with the same symptoms; thus, a kidney biopsy was performed on both siblings. The biopsy findings, which were almost identical, demonstrated mild mesangial proliferation and IgM positivity in the capillary walls. Silver nitrogen staining showed irregularity in the GBM. This was confirmed by an electron microscopic study (Figure 7b). After the second kidney biopsy, enalapril was continued with losartan and her renal function remained stable until August 2010. Her PCr level increased over the following year from 0.96 mg/dL to 7.9 mg/dL, and hemodialysis was started in September 2012, 11 years after her symptoms first appeared. She received a kidney transplant from a living donor in November 2012. Her PCr level at one year after the transplantation was 0.68 mg/gL and she did not have proteinuria or hematuria.

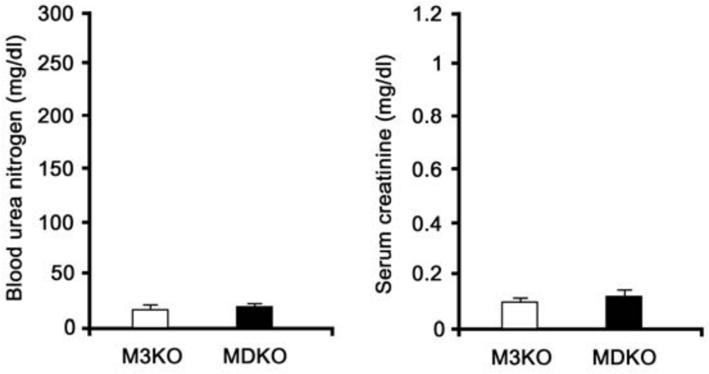

Supplementary Figure S1: We observed no significant differences in the blood urea nitrogen and serum creatinine levels between M3KO and MDKO mice at 7 weeks of age.

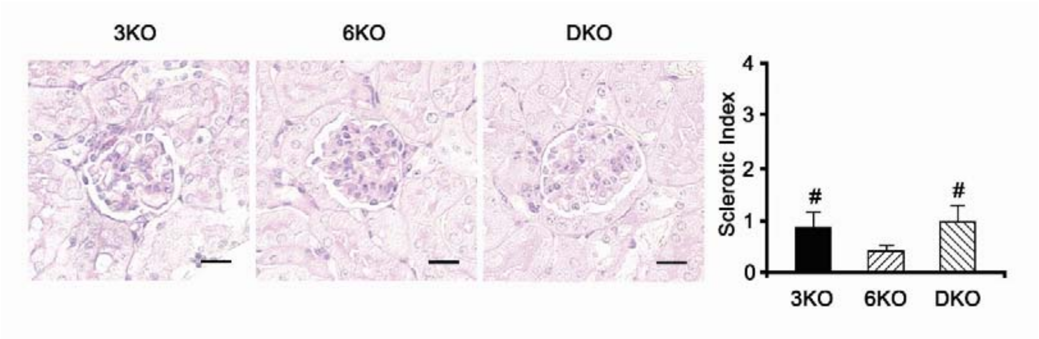

Supplementary Figure S2: The sclerotic indices of 3KO and DKO mice significantly increased in comparison to that of 6KO mice at 7 weeks of age (#*P* < 0.05). Scale bars, 20 μm.

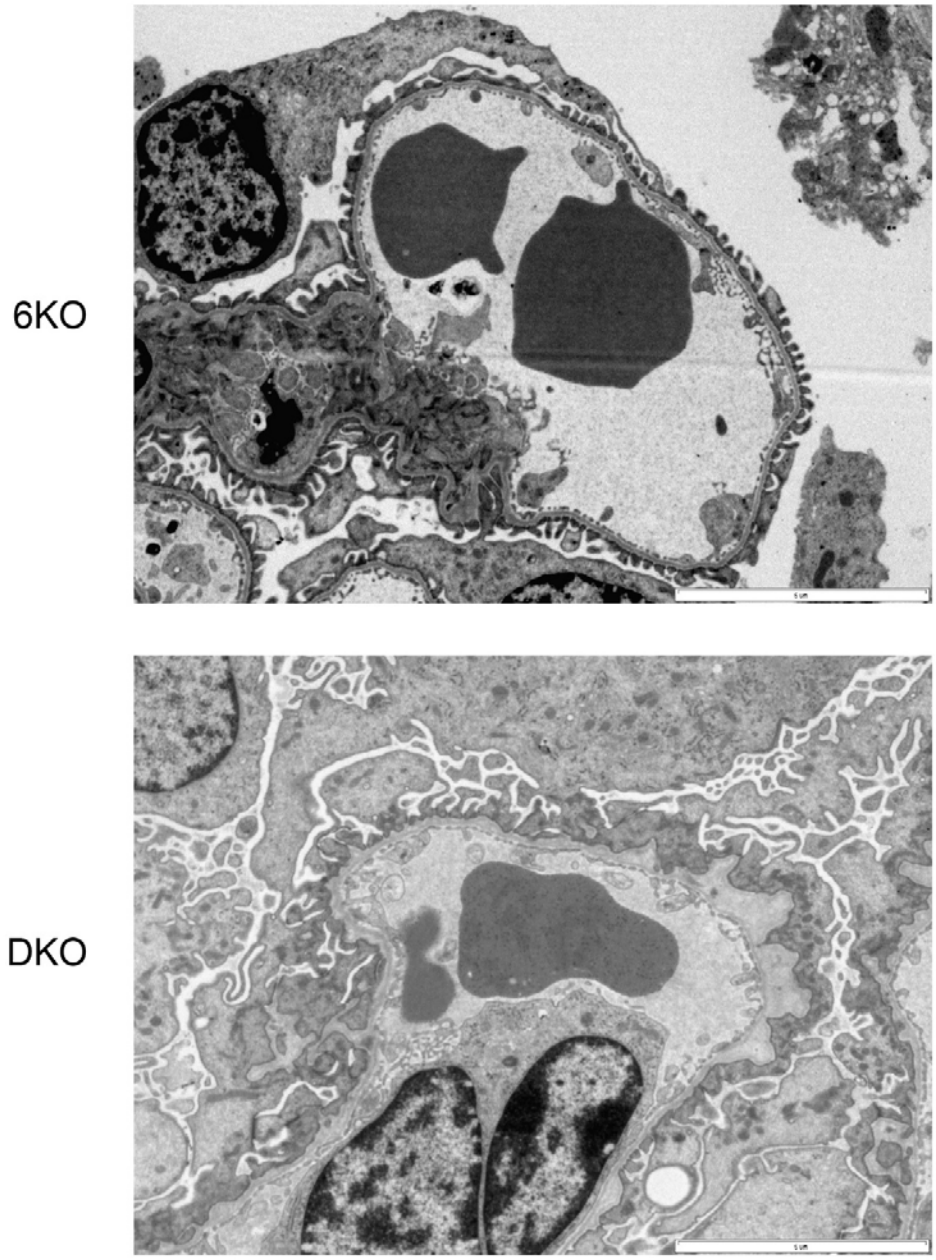

Supplementary Figure S3: An electron microscopic examination at 11 weeks of age showed that the GBM of 6KO mice was unaffected while that of DKO mice exhibited typical changes of AS.

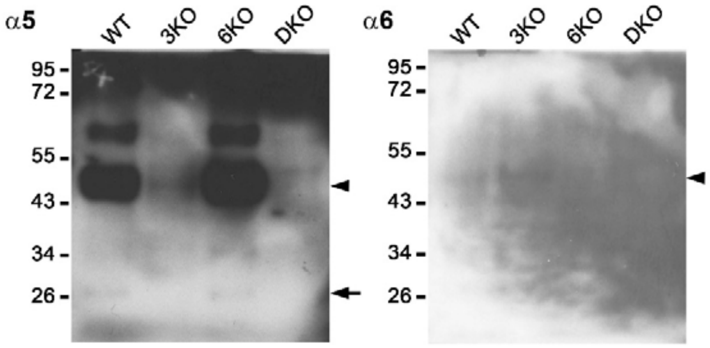

Supplementary Figure S4: The expression of the α5 (IV) and α6 (IV) chains in the RIPA buffer-treated pellets. The dimer of the α5 (IV) chains was detected in the WT and 6KO mice, and it was weakly detected in 3KO mice, whereas the signal was undetectable in DKO mice (arrowhead). The monomer of the α5 (IV) chains was detected in the WT and 6KO mice whereas the signal was undetectable in 3KO and DKO mice (arrow). The dimer of the α6 (IV) chains were weakly detected in the WT and 3KO mice, but it was undetectable in the 6KO and DKO mice (arrowhead).

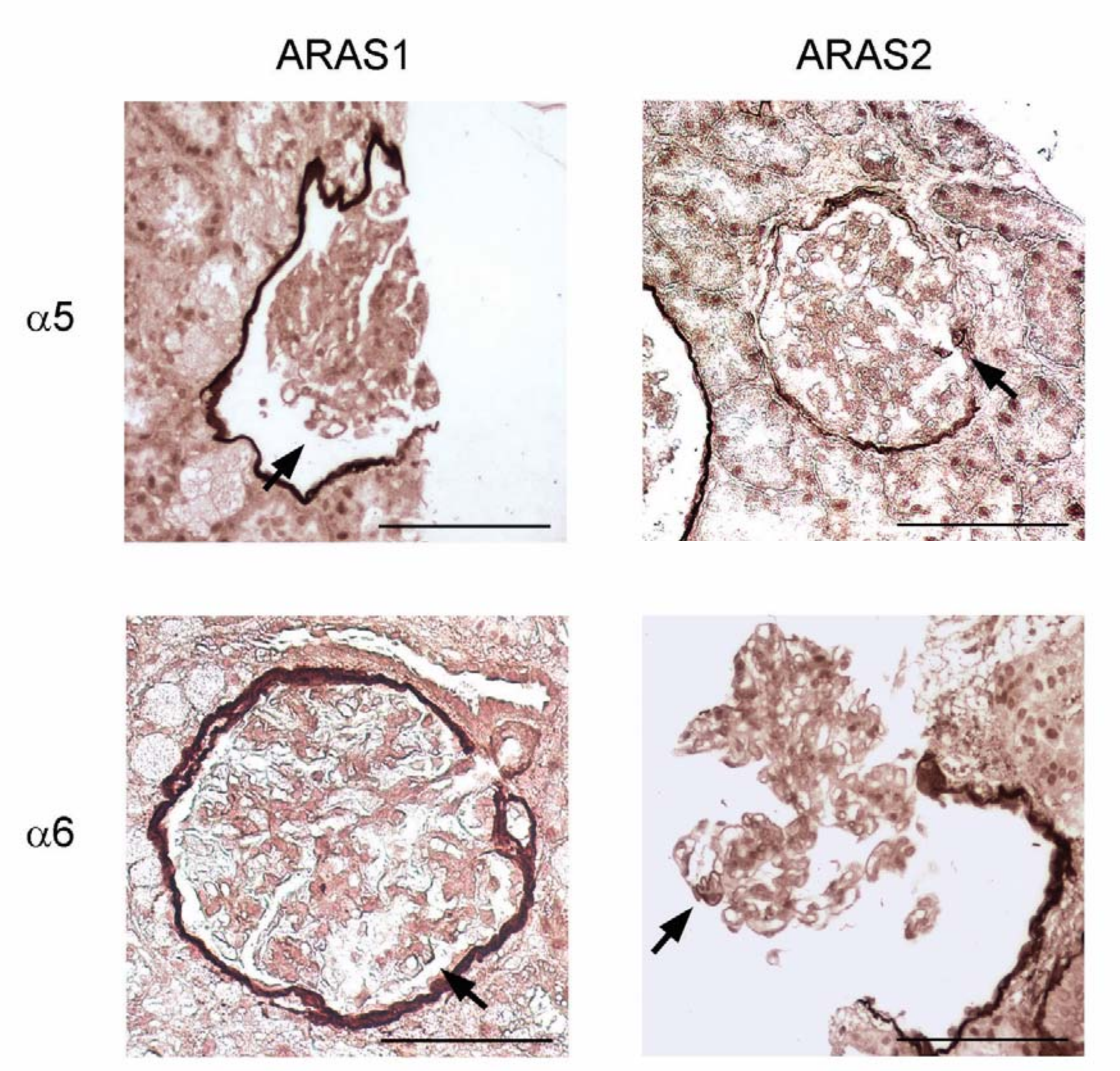

Supplementary Figure S5: α5 and α6 staining of some areas of the GBM was positive in the two patients (arrows).

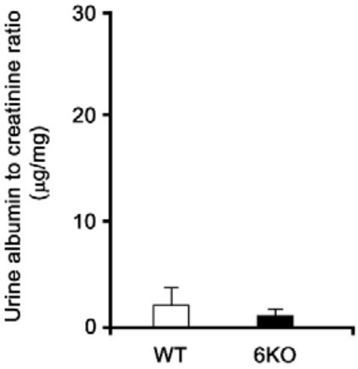

Supplementary Figure S6: We observed no significant differences in the urine albumin to creatinine ration in WT and 6KO mice at 12 weeks of age with a C57BL/6 background. WT (1.7 ± 1.9 µg/mg, n = 5), 6KO (0.7 ± 0.6 µg/mg, n = 5).

Supplementary Table S1: Blood chemistry test.

	BUN (mg/dl)		Cr (mg/dl)	
	7 weeks (n = 10)	11weeks(n = 10)	7 weeks (n = 10)	11 weeks (n = 10)
WT	20.9 ± 4.4	24.5 ± 4.7	0.125 ± 0.018	0.139 ± 0.029
3KO	24.5 ± 2.7	142.2 ± 56.8	0.154 ± 0.027	0.712 ± 0.280
6KO	18.9 ± 2.5	23.7 ± 4.3	0.066 ± 0.037	0.104 ± 0.043
DKO	23.9 ± 4.1	193.6 ± 98.9	0.146 ± 0.018	0.790 ± 0.288
	7 weeks (n = 5)		7 weeks (n = 5)	
M3KO	17.6 ± 5.3		0.106 ± 0.017	
MDKO	19.5 ± 1.6		0.126 ± 0.025	

BUN: blood urea nitrogen, Cr: serum creatinine.

Supplementary Table S2: Morphological evaluation.

	Sclerotic index		Fibrotic index
	7 weeks (n = 5)	11 weeks (n = 5)	11 weeks (n = 5)
WT	not done	0.18 ± 0.10	0.10 ± 0.06
3KO	0.93 ± 0.24	2.78 ± 0.51	3.09 ± 0.32
6KO	0.41 ± 0.14	0.75 ± 0.24	0.11 ± 0.04
DKO	1.00 ± 0.30	2.97 ± 0.60	3.09 ± 0.30

Supplementary Table S3: Real-time reverse transcription polymerase chain reaction of *Col4a1* to *Col4a6*.

	Col4a1	*Col4a2*	*Col4a3*	*Col4a4*	*Col4a5*	*Col4a6*
	11 weeks (n = 3)	11 weeks (n = 3)	11 weeks (n = 3)	11 weeks (n = 3)	11 weeks (n = 3)	11 weeks (n = 3)
WT	2.2 ± 1.0	1.1 ± 0.3	0.7 ± 0.4	0.8 ± 0.2	1.4 ± 0.4	1.3 ± 0.5
3KO	8.4 ± 4.1	4.3 ± 1.2	0.0 ± 0.0	0.9 ± 0.1	2.2 ± 0.3	1.6 ± 0.7
6KO	1.8 ± 0.1	1.2 ± 0.3	0.3 ± 0.1	0.8 ± 0.1	1.5 ± 0.2	0.0 ± 0.0
DKO	7.4 ± 5.9	4.9 ± 3.6	0.0 ± 0.0	1.1 ± 1.1	1.4 ± 1.1	0.0 ± 0.0
